# *Lactobacillus rossiae* strain isolated from sourdough produces putrescine from arginine

**DOI:** 10.1038/s41598-018-22309-6

**Published:** 2018-03-05

**Authors:** Beatriz del Rio, Patricia Alvarez-Sieiro, Begoña Redruello, María Cruz Martin, María Fernandez, Victor Ladero, Miguel A. Alvarez

**Affiliations:** Dairy Research Institute (IPLA-CSIC), Paseo Rio Linares s/n, 33300 Villaviciosa, Spain

## Abstract

This work reports a *Lactobacillus rossiae* strain (*L. rossiae* D87) isolated from sourdough that synthesizes putrescine - a biogenic amine that raises food safety and spoilage concerns - from arginine via the ornithine decarboxylase (ODC) pathway. The *odc* and *potE* genes were identified and sequenced. These genes respectively encode ornithine decarboxylase (Odc), which participates in the decarboxylation of ornithine to putrescine, and the ornithine/putrescine exchanger (PotE), which exchanges ornithine for putrescine. Transcriptional analysis showed that *odc* and *potE* form an operon that is regulated transcriptionally by ornithine in a dose-dependent manner. To explore the possible role of the ODC pathway as an acid stress resistance mechanism for this bacterium, the effect of acidic pHs on its transcriptional regulation and on putrescine biosynthesis was analysed. Acidic pHs induced the transcription of the *odc*-*potE* genes and the production of putrescine over that seen at neutral pH. Further, putrescine production via the ODC system improved the survival of *L. rossiae* D87 by counteracting the acidification of the cytoplasm when the cells were subjected to acidic conditions. These results suggest the ODC pathway of *L. rossiae* D87 provides a biochemical defence mechanism against acidic environments.

## Introduction

Lactic acid bacteria (LAB) play an essential role as biopreservatives in the production of fermented foods, preventing spoilage and contamination by pathogenic microorganisms. LAB are commonly added as starter cultures, but during manufacture and ripening a complex secondary microbiota (non-starter lactic acid bacteria [NSLAB]) develops^1^. Both starter and NSLAB microorganisms contribute towards the production of flavour compounds and the physico-chemical changes that confer the desired organoleptic characteristics sought^2,3^. The metabolic activity of some LAB strains can, however, lead to the production of undesirable or even toxic compounds such as biogenic amines (BAs).

BAs are low molecular weight nitrogenous compounds essential to several physiological functions in humans, e.g., signal transduction at synapses, the immune response, cell growth and gene expression, etc. However, they can accumulate in foods and animal feed via the microbial decarboxylation of certain amino acids^4,5^. The intake of foods containing high concentrations of BAs can produce important adverse effects, such as hypertension, heart palpitations, migraines, nausea and diarrhoea^6^. The most commonly encountered BAs in fermented food are histamine, tyramine and putrescine^4^. Putrescine has been found in high concentration in cheese^7^, sausages^8^, fish and meat products^9^, wine^10^, beer^11^ and cider^12^. Putrescine concentrations can vary widely, but values as high as 900 mg kg^−1^ have been reported^5,6,13,14^. This BA can affect the organoleptic characteristics of food, but when ingested in high concentrations it can lead to increased cardiac output, tachycardia and hypotension. It also potentiates the toxic effect of other BAs^6^ and even participates directly in the promotion of malignancy via its role in the regulation of cell growth and the transformation of cells, as well as indirectly through the production of secondary amines that combine with nitrites to generate carcinogenic nitrosamines^6,15^.

LAB produce putrescine in fermented foods via either the ornithine or agmatine pathway^16,17^. In the ornithine pathway, ornithine is decarboxylated by ornithine decarboxylase (ODC) to form putrescine. In the agmatine pathway, agmatine is deiminated to putrescine via agmatine deiminase (AGDI)^16,18,19^. The AGDI pathway has been shown prevalent in microorganisms isolated from dairy and cider products^12,20^, while microorganisms isolated from wine, meat and sugarcane have been shown to follow the ODC pathway^16^. Certainly, *Lactobacillus mali* strains isolated from cider^21^ and *Lactobacillus brevis* strains isolated from sugarcane^22^ follow this pathway, as do several strains of *Oenococcus oeni* isolated from wine and cider^12,21^. Indeed, the ornithine decarboxylase (Odc) enzymes of *O. oeni* have been extensively characterized^23,24^. In addition to Odc, which is encoded by the *odc* gene, the ODC pathway involves an ornithine/putrescine exchanger (PotE) encoded by *potE*^22^.

The physiological role of amino acid decarboxylative pathways in microorganisms is still uncertain. It may offer a way of producing metabolic energy via an electrogenic amino acid and/or amine antiporter system leading to the generation of proton motive force^25,26^. However, the main function of the amino acid decarboxylative pathways appear to be defence against acidic environments such as those encountered in fermented products^25,27–30^.

This paper reports a *Lactobacillus rossiae* strain (*L. rossiae* D87) - previously isolated from sourdough - to be capable of synthesising putrescine via the ODC pathway. The present work provides a genetic characterization and the transcriptional analysis of the ODC gene cluster and examines the possible role of the ODC pathway as an acid stress resistance mechanism.

## Results

### Production of BAs by *L. rossiae* D87

To check the capacity of *L. rossiae* D87 to produce histamine, tyramine, cadaverine and putrescine, it was grown in De Man, Rogosa and Sharpe (MRS) supplemented with the corresponding precursors (histidine, tyrosine, lysine, and ornithine or agmatine in the case of putrescine) at a final concentration of 2.5 mM. The strain was not able to decarboxylate histidine, tyrosine or lysine (data not shown). However, it was able to produce putrescine from ornithine, although not from agmatine (data not shown), indicating *L. rossiae* D87 follows the ODC pathway.

To monitor the production of putrescine from ornithine over the growth curve, *L. rossiae* D87 was grown in MRS broth and in MRS + Ort (i.e., supplemented with 20 mM ornithine) and the concentrations of arginine, ornithine and putrescine quantified by UHPLC at different times. In the culture supplemented with ornithine, concomitant production of putrescine was observed (Table 1). Surprisingly, the concentration of putrescine was slightly higher than the amount of ornithine added. Indeed, putrescine production was also observed in cultures with no ornithine supplementation. In both cases, the non-expected amount of putrescine detected corresponded to the amount of arginine present in the medium (Table 1). Arginine is not a direct substrate for the production of putrescine. However, many bacteria can follow the arginine deimination (ADI) pathway^31^, in which arginine is deiminated to ornithine with the concomitant production of ATP and NH_4_^+^. To test whether *L. rossiae* D87 could deiminate arginine towards ornithine via the ADI pathway and its further decarboxylation to putrescine via the ODC pathway, cultures supplemented with arginine instead of ornithine were monitored for the production of putrescine. As shown in Table 1, most of the arginine present in the medium (23.4 ± 2.8 mM) was converted into putrescine at 48 h (19.9 ± 5.1 mM), indicating that *L. rossiae* D87 can effectively form putrescine from arginine.

**Table 1:** Arginine [Arg], ornithine [Ort] and putrescine [Put] quantification in supernatants of *L. rossiae* D87 cultures grown in MRS + Ort or MRS + Arg. nd: not detected.

Hours	MRS			MRS + Ort 20 mM			MRS + Arg 20 mM		
	[Arg] mM	[Ort] mM	[Put] mM	[Arg] mM	[Ort] mM	[Put] mM	[Arg] mM	[Ort] mM	[Put] mM
0	3.8 ± 0.1	0.3 ± 0.1	nd	3.7 ± 0.1	21.3 ± 1.7	nd	23.4 ± 2.8	0.2 ± 0.1	nd
4	3.6 ± 0.1	0.2 ± 0.1	nd	3.5 ± 0.4	21.2 ± 1.9	0.4 ± 0.1	22.8 ± 2.9	0.2 ± 0.1	nd
10	2.7 ± 1.5	0.3 ± 0.1	1.2 ± 1.0	2.9 ± 0.5	11.9 ± 4.2	10.1 ± 5.1	21.9 ± 2.8	1.5 ± 0.6	0.3 ± 0.2
17	0.5 ± 0.1	0.2 ± 0.1	3.2 ± 0.5	1.0 ± 0.2	3.2 ± 1.6	15.2 ± 4.7	18.2 ± 2.6	6.2 ± 0.7	1.1 ± 0.6
24	0.2 ± 0.2	0.1 ± 0.1	2.5 ± 0.6	0.3 ± 0.2	2.1 ± 0.7	21.9 ± 3.2	16.9 ± 3.1	5.1 ± 0.6	2.0 ± 0.5
30	0.3 ± 0.1	0.1 ± 0.1	2.7 ± 0.8	nd	0.2 ± 0.1	22.6 ± 2.3	12.1 ± 3.0	6.0 ± 0.9	3.2 ± 1.2
33	0.2 ± 0.1	nd	2.7 ± 0.8	nd	nd	23.7 ± 3.0	7.1 ± 1.8	6.0 ± 0.2	9.1 ± 0.9
48	nd	nd	2.6 ± 1.0	nd	nd	26.7 ± 2.7	0.2 ± 0.1	5.4 ± 1.0	19.9 ± 5.1

### Sequence analysis of the *L. rossiae* D87 ODC cluster

**Figure 1:**
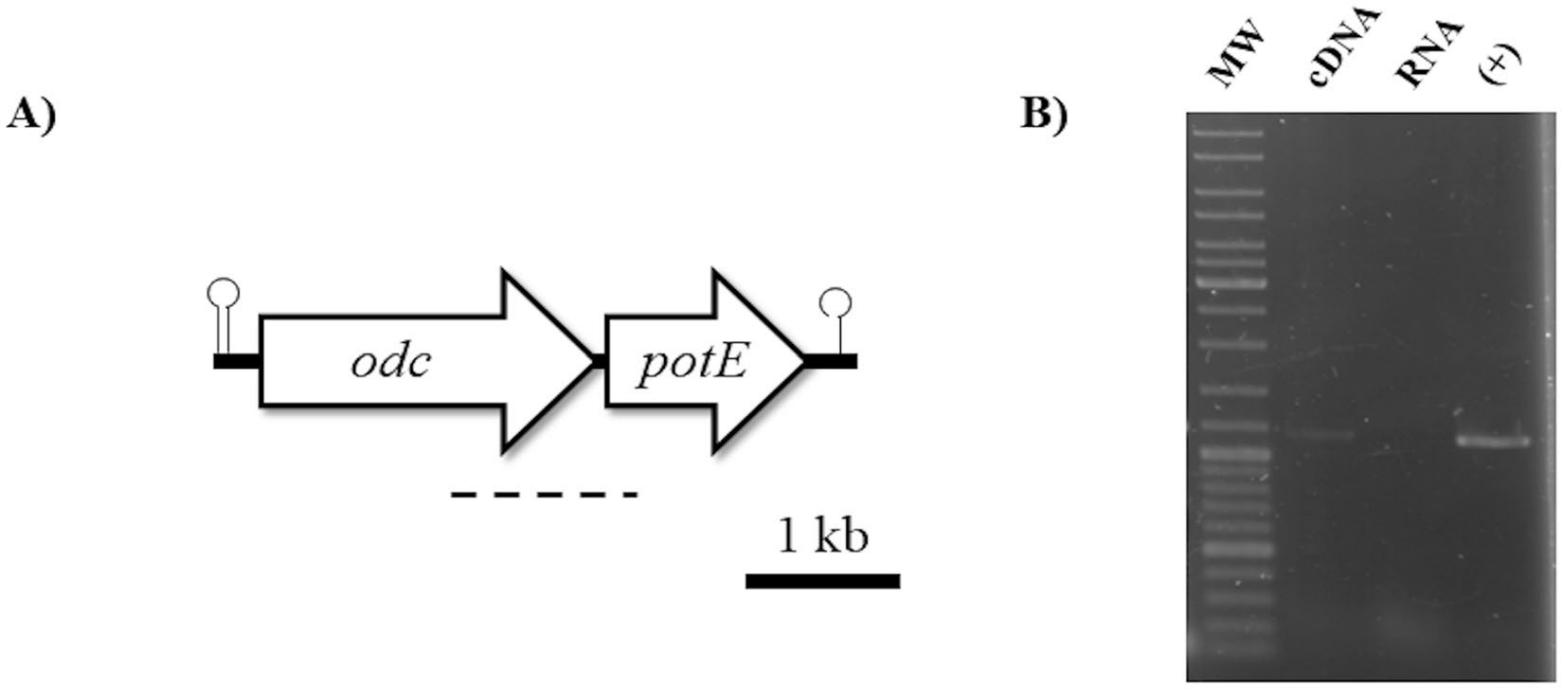
Genetic analysis of the ODC cluster in *L*. *rossiae* D87. (**A**) Diagram of the genetic organization of the ODC gene cluster of *L. rossiae* D87. Arrows indicate the position and direction of the *odc* and *potE* genes. Lollipop: putative transcriptional terminators. Dotted line: position of the RT-PCR fragment amplified in panel (B). (**B**) RT-PCR amplification of the intergenic region *odc*-*potE* in *L. rossiae* D87. Negative controls (RNA) were performed using the same RNA sample but without reverse transcriptase; positive controls (+) were performed with chromosomal DNA. MW: molecular weight marker (GeneRuler DNA ladder mix [Fermentas]).

To identify the genes involved in the production of putrescine, a Polymerase Chain Reaction (PCR) strategy using the primers previously described for the detection of the *odc* gene ODC3/ODC16^32^ was followed. A single band of the expected size was obtained (data not shown). The fragment was sequenced and showed similarity to *odc* genes. To obtain the complete sequence of the putrescine-production-associated genes, the unknown DNA sequences adjacent to the *odc* fragment gene were examined in both the 5′ and 3′ directions using a combination of reverse PCR and PCR amplification with primers based on the sequence of ODC clusters available in databases. A 4165 bp nucleotide sequence was finally obtained (GenBank accession no. KT020759). Sequence analysis showed the ODC cluster to be composed of two genes with the same orientation: *odc* and *potE* (Fig. 1A). The cluster was enclosed between two putative rho-independent terminators (ΔG = −13 kcal upstream of *odc* and ΔG = −9.8 kcal downstream of *potE*). A conserved ribosome binding site (RBS) was identified 7 nt upstream of the *odc* start codon and 8 nt upstream of that of *potE*. No clear promoter consensus sequence was found upstream of *odc* or *potE*.

During this work, an internal fragment of another *odc* gene from a different *L. rossiae* strain was deposited in the GenBank database (Accession no. HQ141619). Its nucleotide sequence and that of *L. rossiae* D87 were 96% identical. The *L. rossiae* D87 *odc* gene was also similar to *odc* from *Lactobacillus brevis* IOEB 9906 (77%), *O. oeni* BIFI-83 (74%) and *Lactobacillus saerimneri* 30a (68%), and encoded a protein of 725 amino acids with a predicted molecular weight of 82 kDa. The deduced Odc protein showed a typical pyridoxal 5′-phosphate binding pocket in its extreme N-terminal, followed by the catalytic centre.

*potE* was similar to other *potE* genes adjacent to *odc* in *L. brevis* IOEB 9906 (71%) and *O. oeni* BIFI-83 (72%). This gene encodes a protein, PotE, of 437 amino acids with a predicted molecular weight of 46.5 kDa. PotE was found to have 12 transmembrane domains, supporting its role as an ornithine/putrescine membrane antiporter, like that of *L. brevis* IOEB 9906^22^.

**Figure 2:**
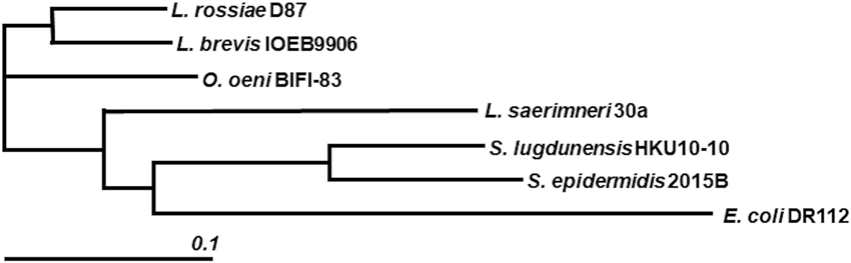
Phylogenetic analysis of Odc proteins. Phylogenetic tree constructed at The European Bioinformatics Institute (EMBL-EBI), based on a ClustalW alignment and clustered by Neighbour-joining without distance corrections. Odc amino acidic sequences were from *Lactobacillus rossiae* D87 (KT020759), *Lactobacillus brevis* IOEB 9906 (AFC60624), *Lactobacillus saerimneri* 30a (WP_009553942), *Oenococcus oeni* BIFI-83 (CAG34069), *Staphylococcus lugdunensis* HKU10-10 (ADU52586), *Staphylococcus epidermidis* 2015B (YP_009088105) and *Escherichia coli* DR112 (AAA62785).

The phylogenetic tree (Fig. 2) constructed - based on the clustalW alignment of those ODC proteins that have been described involved in the production of putrescine^22^ - showed the ODCs of LABs to be closely related (except that of *L. saerimneri* 30a).

### *odc* and *potE* are cotranscribed as a monocistronic mRNA

Before starting the transcriptional analysis of factors affecting the expression of the ODC cluster, the transcriptional profile of the ODC cluster was determined. cDNA from total RNA of cultures grown in MRS + Ort was used in Reverse Transcription Polymerase Chain Reaction (RT-PCR) amplifications with a pair of primers (Table 2) designed to amplify the intergenic region between *odc* and *potE*. A PCR band of the expected size was obtained (Fig. 1A,B) showing that these genes are cotranscribed.

**Table 2:** Primers used in this study.

**Primer**	**Function**	**Sequence (5′-3′)**	**Reference**
pA	*16S rRNA* amplification	AGAGTTTGATCCTGGCTCAG	^52^
pH	*16S rRNA* amplification	AAGGAGGTGATCCAGCCGCA	^52^
ODC3	ODC cluster detection	GTNTTYAAYGCNGAYAARACNTAYTTYGT	^32^
ODC16	ODC cluster detection	TACRCARAATACTCCNGGNGGRTANGG	^32^
ODC5	Reverse PCR and sequencing	CAACGCTTATATGCAATATGCTTCAA	This work
ODC6C	Reverse PCR and sequencing	GTAAACAGATTTATGGTTATTACGATC	This work
ODC3C	Sequencing	GTTCCACCTAAAACAGCTATTGGAAC	This work
ODC4C	Sequencing	GTTCCAATTTGAGCAAGGTTAGTTGGC	This work
ODC6	Sequencing	ACTGTGCCAAGAGTTACATGACTTC	This work
ODC7	Sequencing	GAACCATGTCAATTGTTTCGTGGGTTG	This work
ODC87seq	Sequencing	CGCTAAGTTAGCCGCATCAAATGC	This work
QODCD7F	RT-PCR forward and RT-qPCR *odc* forward	GTTCAGACGGGTGAATATGAAGACTTT	This work
PotQLRR	RT-PCR reverse	GTTCATAACCGTAGCCAACCAAA	This work
QODCD7R	RT-qPCR *odc* reverse	CCGTGTTCACGCAAATAGTTAGCT	This work
ROSQ16F	RT-qPCR reference gene	CCGCCTGCACTCCCTTT	This work
ROSQ16R	RT-qPCR reference gene	CGTAGGTGGCAAGCGTTATCC	This work

### The expression of *odc* is induced by ornithine and acidic pH

**Figure 3:**
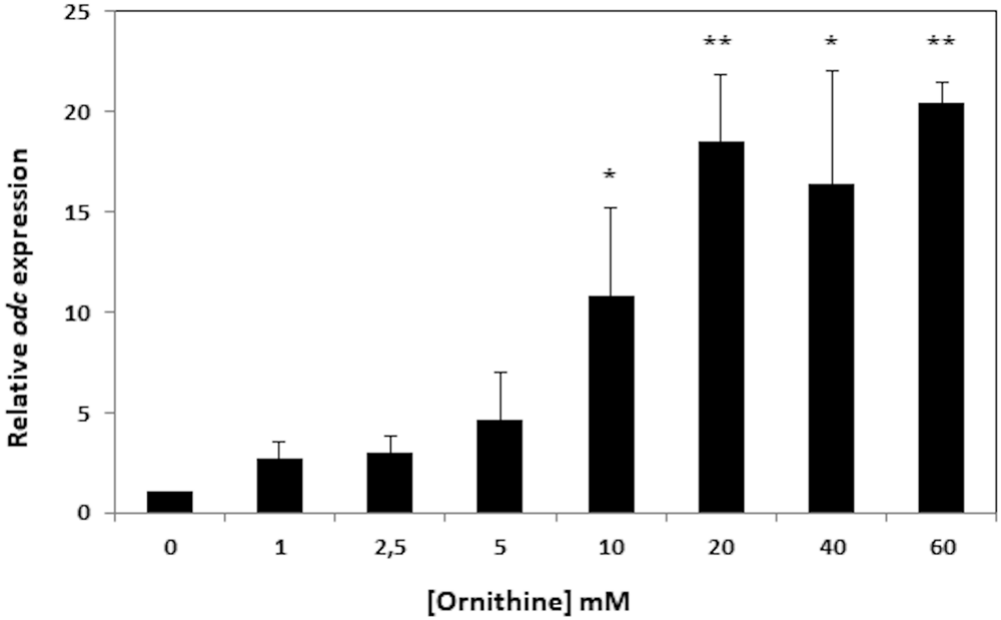
Influence of increasing ornithine concentration on *odc* expression determined by RT-qPCR. Cells were grown on CDM supplemented with 0, 1, 2.5, 5, 10, 20, 40 or 60 mM ornithine, and samples collected at the mid-exponential phase of growth. All samples were normalized to the total RNA content using the *16S rRNA* gene as a reference. The expression of *odc* in absence of ornithine (0) was normalized to 1 and used as the reference condition.

The influence of ornithine on *odc* expression was examined by Reverse Transcription quantitative Polymerase Chain Reaction (RT-qPCR) in CDM supplemented with different concentrations of this compound (Fig. 3). Since *odc* and *potE* were found to be cotranscribed (Fig. 1), only the expression of *odc* was quantified by RT-qPCR. As shown in Fig. 3, the presence of ornithine induced *odc* expression in a concentration-dependent manner.

**Figure 4:**
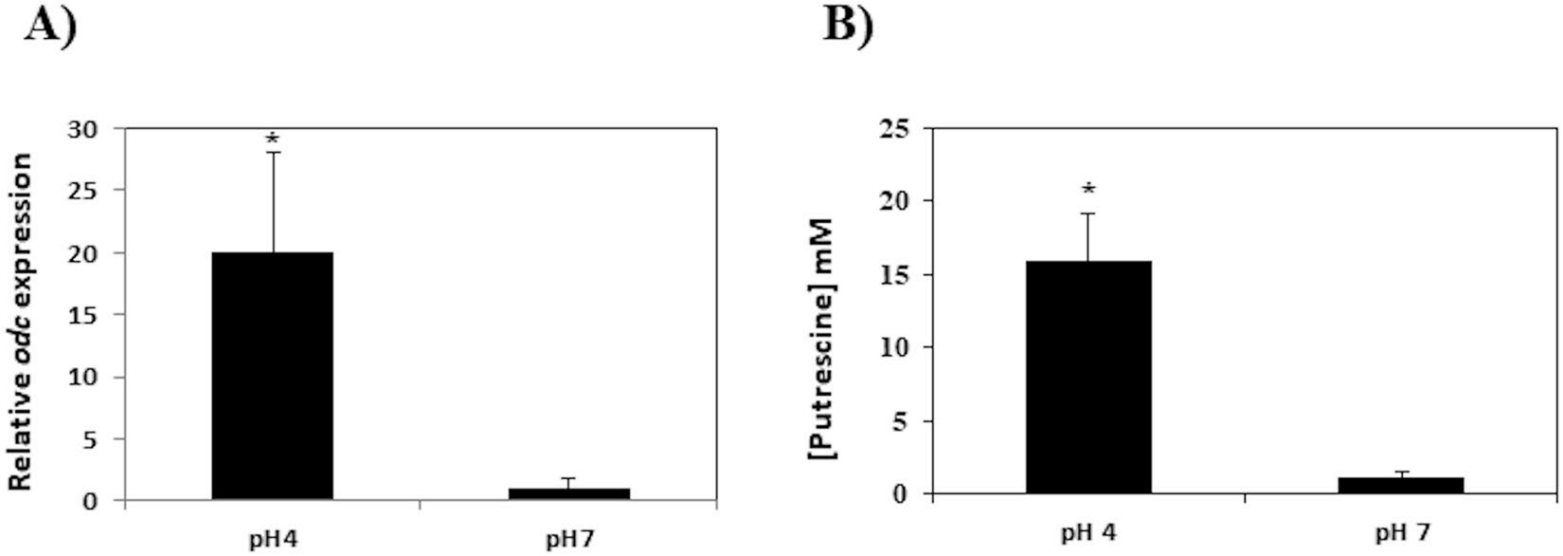
Influence of pH on *odc* transcription and putrescine production. (**A**) Influence of pH (4 *vs* 7) on *odc* transcription in *L. rossiae* D87 grown in MRS + Ort. Expression was normalized to the total RNA content using the *16S rRNA* gene as a reference. The expression at pH 7 was normalized to 1 and used as the reference condition. (**B**) Influence of pH on putrescine production. Cells were grown in CDM adjusted to pH 4 or pH 7 and harvested at the mid-exponential phase of growth. pH was measured and a variation of <0.5 observed. Putrescine concentration in the supernatants was quantified by UHPLC. Values shown are the means of three replicates; standard deviations are indicated by bars. Asterisks indicate statistically significant differences (*p < 0.05, **p < 0.01) as determined by the Student *t* test.

The influence of acidic pH on *odc* expression was also examined. As shown in Fig. 4A, the relative expression of *odc* gene at pH 4 was about 19 times that recorded at pH 7. This indicates that acidic pH induces *odc* expression in *L. rossiae* D87. Accordingly, the production of putrescine increased about 10 times (Fig. 4B).

### Effect of putrescine production on bacterial growth and medium acidification

**Figure 5:**
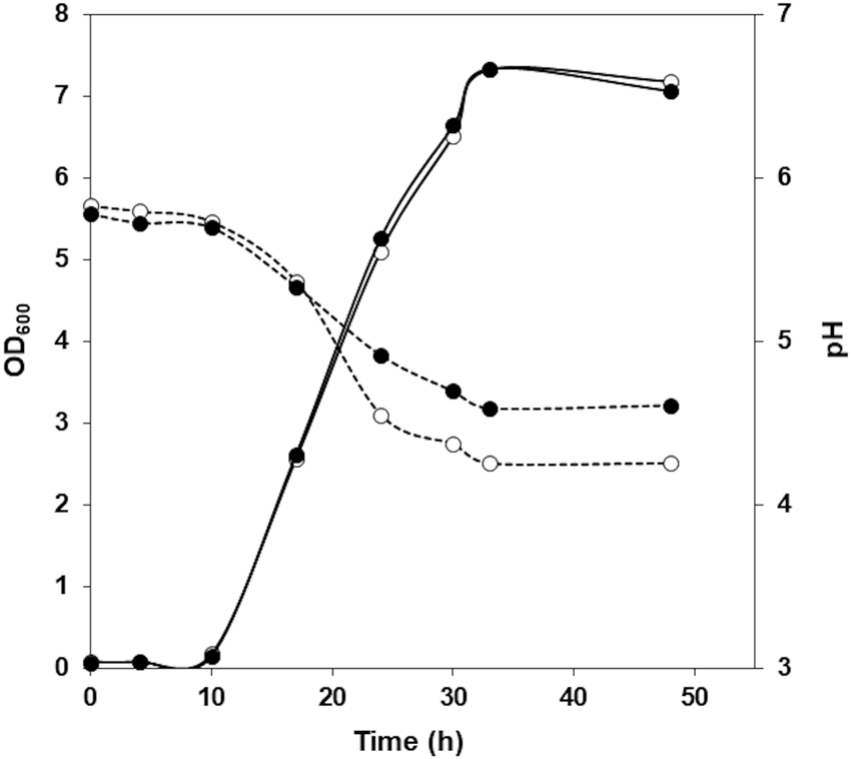
Influence of ornithine supplementation on *L. rossiae* D87 growth. Growth curve of *L. rossiae* D87 in MRS (*empty circles*) and MRS + Ort (*filled circles*). Bacterial growth (*continuous line*) determined by measuring the absorbance at 600 nm (OD_600_), and the pH of the medium (*dotted line*), were monitored for 48 h.

To determine whether the production of putrescine offers any advantage to *L. rossiae* D87 in terms of growth, the OD_600_ and culture pH in MRS and MRS + Ort were monitored (Fig. 5). No differences in growth were observed, although the acidification of the culture media was less strong in the presence than in the absence of ornithine (pH 4.6 compared to pH 4.2). This indicates that the decarboxylation of ornithine to putrescine might play a role in acid resistance.

### Putrescine production improves cell survival under acidic conditions

**Figure 6:**
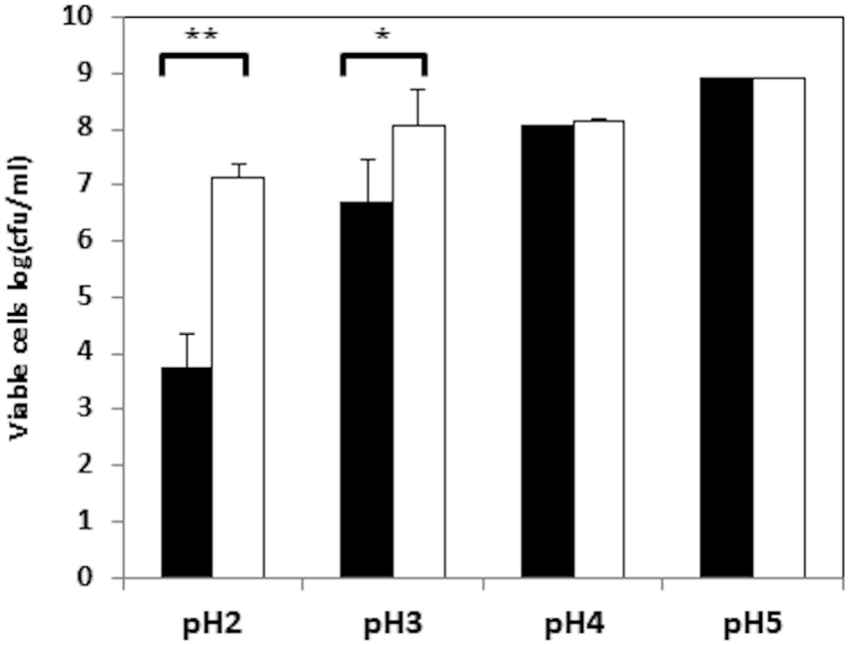
Cell survival after acidic pH challenge. Cells were grown in MRS or MRS + Ort and harvested at mid-exponential growth. The cells were then resuspended in PBS buffer (*filled columns*) or PBS + Ort (*empty columns*) and adjusted to the pH indicated. Cell viability was measured after 2 h. Each value represents the mean of three independent experiments. Differences in survival in the presence of ornithine were tested against the control using the Student *t* test (*p < 0.05, **p < 0.01).

The viability of *L. rossiae* D87 under acidic stress was evaluated in resting cells. As shown in Fig. 6, this strain was highly resistant to acid challenge. Little lethality was seen after exposure of the cells to pH 4 for 2 h, although it increased at pH 3 and especially at pH 2. The presence of ornithine significantly improved cell survival at pH 3 (*p* < 0.05) and particularly at pH 2 (by four orders of magnitude; *p* < 0.01) (Fig. 6). Thus, putrescine production via ornithine decarboxylation improves the fitness of *L. rossiae* D87 under acidic conditions.

### Putrescine production via ornithine decarboxylation counteracts acidification of the cytosol in acidic environments

**Figure 7:**
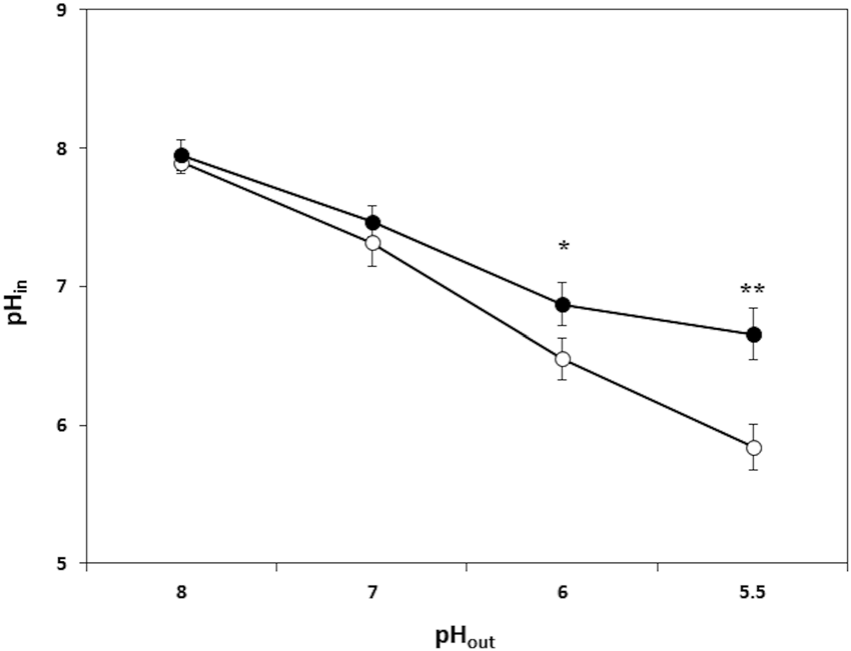
Influence of ornithine in internal pH. Variation in the intracellular pH (pH_in_) at different (8, 7, 6, and 5.5) extracellular pHs (pH_out_) measured using a cFSE probe in resting cells of *L. rossiae* D87 grown in the absence (*empty circles*; control condition) or presence of 20 mM ornithine (*filled circles*). Asterisks indicate statistically significant differences compared to control cultures (*p < 0.05; **p < 0.01; Student *t* test).

It has been suggested that the mechanism underlying the resistance to acid conferred by the production of putrescine via the ODC pathway in LAB is exerted by maintaining the pH of the cytosol^28^. Changes in the cytosolic pH of *L. rossiae* D87 were therefore monitored at external pH values ranging from 8.0 to 5.5 in the absence/presence of ornithine (10 mM). Figure 7 shows that in the absence of ornithine, the internal pH dropped as the external pH decreased. When ornithine was present, a drop in the internal pH was also observed as the external pH is reduced, but the gradient was much shallower than in the absence of ornithine. Between pH_out_ 6 and 5.5, the internal pH values in the presence of ornithine were about 1 pH unit higher than in its absence (pH_in_ 6.7 and 5.8 respectively). This indicates that the production of putrescine via ornithine decarboxylation in *L. rossiae* D87 counteracts the acidification of the cytosol produced by exposure to an acidic pH challenge, and that the effect is greater the lower the external pH.

## Discussion

Putrescine is one of the most commonly encountered BAs in fermented food products^33–35^. Several LAB that produce putrescine from agmatine have been identified and characterized^12,16^; however, far fewer ODC + strains have been described. This work reports *L. rossiae* D87, isolated from sourdough, to be ODC + , increasing the spectrum of species able to produce putrescine from ornithine.

The two possible substrates for the biosynthesis of putrescine - agmatine and ornithine - come from the same amino acid, arginine. While agmatine is the product of arginine decarboxylation, ornithine is the product of its deimination. Putrescine is thus synthesized from arginine by two different routes, each requiring two steps^17^. However, it is important to note that these two steps are not usually performed by the same bacterial species and, therefore, cooperation between different species is usually required for putrescine to accumulate in foods. Remarkably, *L. rossiae* D87 appears able to perform both of the steps required to produce putrescine from arginine via the ODC pathway –the deimination of arginine to ornithine and its further decarboxylation to putrescine. As far as we know, *Weisella halotolerans* W22 is the only other microorganism equipped to undertake both these steps^36^. *L. rossiae* D87 is the first putrescine producer isolated to have been from sourdough, a food in which arginine is available.

*L. rossiae* has been first isolated from sourdough by Corsetti *et al*. in 2005^37^, and it is now well known that it is widely distributed and present in this fermented food^38^. However, the capability of *L. rossiae* sourdough strains to produce BAs has not been previously shown, only one strain isolated from a wine starter has been described as histamine producer^39^. In this work, we describe for first time a strain of *L. rossiae* with capability to produce the BA putrescine. Moreover, the ability of *L. rossiae* D87 to undertake both steps from arginine to putrescine constitutes a property that naturally increases the risk of putrescine accumulation.

This strain could, therefore, easily produce putrescine during the fermentation process. Although the presence of BAs - including putrescine - has not been extensively studied in sourdough, some authors have reported the presence of BAs in sourdoughs made from different flour types^35,40^. The potential presence in sourdough of BA-producing microorganisms other than *L. rossiae* D87 described cannot, therefore, be ruled out.

Since the first description of an ornithine decarboxylation system in the LAB *L. saerimneri* 30a^41^, few new data on the ornithine decarboxylases of this bacterial group have been collected^16^. The ODC of *L. saerimneri* 30a appears to be atypical of those seen in LAB. As shown in Fig. 2, it is more similar to that of microorganisms such as *Staphylococci* than to those of other LAB, including *L. rossiae* D87. In addition, *L. saerimneri* 30a has a unique genomic organization in which *odc* does not have an adjacent specific transporter gene. In contrast, it has a three-component decarboxylase system that, in addition to the *odc* gene, has a lysine decarboxylase gene (*aadc*) and a promiscuous amino acid-amine transporter gene (*aat*)^42^. Phylogenetic tree analysis grouped the *L. rossiae* D87 Odc with the other LAB Odc proteins, suggesting they have a common origin. In *O. oeni* and *L. brevis*, this cluster was acquired through horizontal gene transfer^28,43^, but further work is needed to determine if this is also the case in *L. rossiae* D87.

Transcriptional studies were performed as a first step in the physiological characterization of the ODC pathway in *L. rossiae* D87. *odc* and *potE* were found to be cotranscribed in a single mRNA (Fig. 1A,B). As far as we know, there are no other data available on transcriptional studies of the ODC system in LAB. In Gram-negative bacteria, however, these genes are also cotranscribed and form an operon^44,45^. No previous studies have been performed on the factors that regulate the expression of the ODC system genes in LAB. The present results show that substrate availability and an acidic pH enhances *odc* expression. Both conditions play a determining role in the expression of other amino acid decarboxylases in LAB, such as those involved in the decarboxylation of tyrosine^29,46^ and histidine^47,48^. No putative regulatory gene has been found close to the ODC system that might be a responsible for the control gene expression. The expression of the ornithine decarboxylase gene (*speF*) in *E. coli* is similarly induced by acidic pH and ornithine^45^, and no regulatory gene has been identified either. It has been suggested that RNase III might be involved in the upregulation of the operon^45^.

Putrescine production by ornithine decarboxylation had no effect on *L. rossiae* D87 growth; the same OD was reached in MRS and MRS supplemented with ornithine (Fig. 5). Amino acid decarboxylation has been proposed as a means of obtaining metabolic energy through an electrogenic amino acid and/or amine antiporter system leading to the generation of proton motive force^25,26^. However, physiological studies on other decarboxylation systems involved in BAs production in enterococci or streptococci indicate them not to have a major impact on cell growth^29,47,49^. Instead, the production of putrescine by ornithine decarboxylation seems to play a role in acid resistance. As shown in Fig. 5, cell cultures grown in the absence of ornithine reach a more acidic pH than those supplemented with ornithine. In addition, the expression of *odc* in *L. rossiae* D87 is enhanced at acidic pH, with a concomitantly greater production of putrescine observed (Fig. 4A,B). This might be related to its role in protection against acid stress. The increased survival of *L. rossiae* D87 at pH 3 and pH 2 in the presence of ornithine supports this idea (Fig. 6). It has been shown that the mechanism by which amino acid decarboxylation protects cells against acidic environments is mediated by the control of the internal pH^28,29^, and the present data appear to confirm this. Ornithine decarboxylation reduced the acidification of the intracellular pH at different external pHs (Fig. 7), more effectively so as the external pH fell.

In conclusion, *L. rossiae* D87 is a putrescine-producing microorganism found in sourdough that appears to produce this BA as a defence against the acidic pH of this fermented food. In addition to their negative effects on the organoleptic characteristics of the final product, putrescine-producers may thus pose a potential health risk to consumers, suggesting such foods should be checked for their putrescine content.

## Methods

### Bacterial strains and culture conditions

*L. rossiae* D87, isolated from sourdough was grown at 30 °C without aeration in De Man, Rogosa and Sharpe (MRS) broth (Oxoid, UK).

To test this organism’s capacity to produce BAs it was grown in the same medium supplemented with either histidine, tyrosine, lysine, ornithine or agmatine (final concentration 2.5 mM) as previously described^50^. Where indicated, ornithine or arginine (Sigma-Aldrich, Spain) was added to the medium at different concentrations. When needed, the final pH of the medium was adjusted to pH 2, 3, 4 or 5 with HCl. When necessary, viable cells were counted by pour-plating 10-fold serial dilutions of cell cultures on MRS-agar plates. To study the influence of ornithine on the production of putrescine and the transcriptional activity of *odc*, the strain was grown at 30 °C in the chemically defined medium (CDM) described by Looijesteijn and Hugenholtz^51^.

### Nucleic acid extraction

Total DNA of *L. rossiae* D87 grown in MRS broth was extracted using the Genomic DNA Purification Kit (Sigma-Aldrich) according to the manufacturer’s recommendations. RNA was extracted from 2 ml of cell cultures at mid-exponential growth, following centrifugation in a refrigerated benchtop microcentrifuge (Eppendorf, Germany) running at maximum speed. Total RNA was extracted using TRI reagent (Sigma-Aldrich) as previously described^46^. To eliminate any contaminating DNA, 2 μg of total RNA samples were treated with 2U of DNAse I (Fermentas, Lithuania) for 2 h at 37 °C. The absence of contaminating DNA was checked via reverse transcription quantitative Polymerase Chain Reaction (RT-qPCR) using primers that amplify the 16*S rRNA* gene, ROSQ16F and ROSQ16R (Table 2). Total RNA concentration was determined in an Epoch Microplate Spectrophotometer (BioTek, USA).

### DNA manipulation

*L. rossiae* D87 was identified at the species level by partial amplification and sequencing of the 16*S rRNA* gene. PCR was performed using total DNA as a template (1 ng), the universal primers pA and pH^52^, and 5PRIME Taq DNA polymerase (5 PRIME GmbH, Germany), following the manufacture’s instructions, in an iCycler thermocycler (Bio-Rad, Spain). The PCR programme consisted of 5 min at 95 °C followed by 35 cycles of amplification (30 s at 95 °C, 30 s at 55 °C, and 90 s at 68 °C) and a final extension step of 10 min at 68 °C. The amplified fragments were then purified using the ATP™ Gel/PCR Extraction Kit (ATP TM Biotech Inc., Taiwan) and sequenced at Macrogen (The Netherlands). The resulting sequences were assembled and deposited in the GenBank database (accession number MG149553) and compared with the eubacterial 16*S rRNA* gene sequences available in that database, and those in the EMBL database, using BLAST software^53^.

The primer pair ODC3 and ODC16 was used to PCR-amplify a 1.49 kb fragment within *odc* from *L. rossiae* D87 following the method described by Marcobal, *et al*.^32^. PCR products were purified and sequenced as described above, and used together with the ODC cluster sequences available in public genome databases to design primers (Table 2) for progressive “genome walking” sequencing analysis combined with reverse PCR. For this, total DNA from *L. rossiae* D87 was digested with *Eco*RI or *Hind*III, diluted, and ligated with T4 DNA ligase (Fermentas) following the manufacturer’s instructions. Ligated fragments were used as templates, directly and 10-fold diluted, for inverse PCR with the primers ODC5 and ODC6C (Table 2). The various PCR fragments generated with standard and reverse PCR were purified and sequenced at Macrogen, and assembled using Vector NTI Advance software, v.9.1 (Invitrogen, USA). Sequence analysis was performed using the University of Wisconsin Genetics Computer Group software package. BLAST and BLASTP programs were used to determine the similarities of the deduced amino acid sequences to those present in databases. Multiple alignment was performed using ClustalW2 at EBI (http://www.ebi.ac.uk/Tools/msa/clustalw2/) and phylogenetic trees obtained using TreeView v.1.6.6. The transmembrane elements of the predicted proteins were examined using the TMHMM tool (http://www.cbs.dtu.dk/services/TMHMM/; Danish Technical University, Denmark). The nucleotide sequence of the ODC cluster reported in this paper is available at GenBank (http://www.ncbi.nlm.nih.gov) under accession number KT020759.

### Reverse transcription PCR (RT-PCR) analysis

cDNA was synthesized from 2 μg of total RNA extracted from *L. rossiae* D87 cells grown in MRS, and MRS supplemented with ornithine (10 mM), using the iScript™ cDNA Synthesis Kit (Bio-Rad, UK) following the manufacturer’s recommendations. PCR was performed as described above but using 2 μl of the cDNA preparation as a template and 0.4 μM of each primer, i.e., QODCD7F and PotQLRR (Table 2). Amplifications were performed over 35 cycles (94 °C for 30 s, 50 °C for 25 s, and 68 °C for 1 min), and the products analyzed in 0.8% agarose gels in TAE buffer. The absence of contaminating DNA was checked by PCR performed under the conditions described above, using total RNA as a template.

### RT-qPCR analysis

RT-qPCR was performed using the SYBR Green PCR Master Mix Kit (Applied Biosystems, UK) employing the primer pair QODCD7F and QODCD7R for the amplification of *odc*, and primer pair ROSQ16F and ROSQ16R for the amplification of *16S rRNA* (Table 2). All reactions were performed in 20 μl volumes, including 1 μl of cDNA (obtained as described above) as a template, 900 nM of each primer, and 10 μl of SYBR Green PCR Master Mix (which contains ROX as a passive reference dye) (Applied Biosystems). Amplification and detection were performed using an ABI Prism Fast 7500 sequence detection system (Applied Biosystems) under standard running conditions. The cycle thresholds (Ct) used for comparison were those automatically assigned by the thermocycler software (7500 Software v2.0.4, Applied Biosystems). Relative gene expression was calculated using the ΔΔCt comparative method, employing *16S rRNA* as a housekeeping gene^54^.

### Analysis of BAs production by UHPLC

The production of BAs, including putrescine, in *L. rossiae* D87 supernatant cultures - prepared as previously described^47^ - under each set of experimental conditions (MRS or CDM supplemented with different substrate, histidine, tyrosine, lysine, agmatine, arginine or ornithine concentrations) was examined by UHPLC. Cultures were centrifuged at 2250 *g* for 10 min, and 1 ml of the resulting supernatant filtered through a 0.2 μm Supor membrane (Pall, UK) and analysed for the presence of putrescine. Briefly, supernatants (100 μl) were derivatized using diethyl ethoxymethylenemalonate (Sigma-Aldrich) and injected onto a Waters Acquity UPLC^TM^ BEH C_18_ 1.7 μm column in an H-Class Acquity UPLC system (Waters, USA) coupled to a photodiode array detector at 280 nm and controlled by Empower 2 software (Waters). The gradient and detection conditions were those previously described^55^.

### Resistance to acidic pH

To test the resistance of *L. rossiae* D87 to acid stress, mid-exponential cultures of *L. rossiae* D87 grown in MRS were harvested by centrifugation and resuspended at a final concentration of 10^8^ cfu ml^−1^ in phosphate-buffered saline (PBS) adjusted to pH 2, pH 3, pH 4 or pH 5, and in the same buffers but supplemented with 20 mM ornithine, for 2 h. After this time, cell survival was evaluated by counting.

### Measurement of intracellular pH

Cytosolic pH measurements were performed using carboxyfluorescein succinimidyl ester (cFSE, Sigma-Aldrich) (an internally conjugated fluorescence pH probe) following a previously described protocol^29^ with minor modifications. Briefly, instead of tyramine, 10 mM ornithine was added before the quantification of fluorescence. The fluorescence intensities of 200 μL cells were measured for 15 min (intervals of 0.5 s) in a Cary Eclipse fluorescence spectrophotometer (Varian Inc., USA) (excitation wavelengths 440 nm and 460 nm, emission wavelength 525 nm)^56^. Background fluorescence levels were assessed by measuring non-fluorescent control cells; these values were subtracted from the fluorescence results. The cytosolic pH values were determined from the ratio of the fluorescence signal at 440/490 nm taken from a calibration curve constructed using buffers at pH 4.5–8.0, after equilibrating the internal (pH_in_) and external (pH_out_) pH with 0.1% triton^57^. The value given for each condition is the average of measurements for three independent replicates.

### Statistical analysis

Means ± standard deviations were calculated for at least three independent replicates and compared using the Student *t* test. Significance was set at *p* < 0.05.

## Electronic supplementary material

Supplementary material
