# Supplementary material for: Lactobacillus rossiae strain isolated from sourdough produces putrescine from arginine
Source: Sci Rep. 2018 Mar 5;8:3989. doi: 10.1038/s41598-018-22309-6 (PMC5838238; doi:10.1038/s41598-018-22309-6)

Lactobacillus rossiae strain isolated from sourdough produces putrescine from arginine

Beatriz del Rio, Patricia Alvarez-Sieiro, Begoña Redruello, María Cruz Martin, María Fernandez, Victor Ladero* and Miguel A. Alvarez

Supplementary material Figure 1. Original image shown in Figure 1B. The image was acquired with a G-box (syngene) and stored in jpg file format. The four first lanes were not related to the experiment described in the manuscript and were removed. Gel was cropped in powerpoint to shown only related bands.

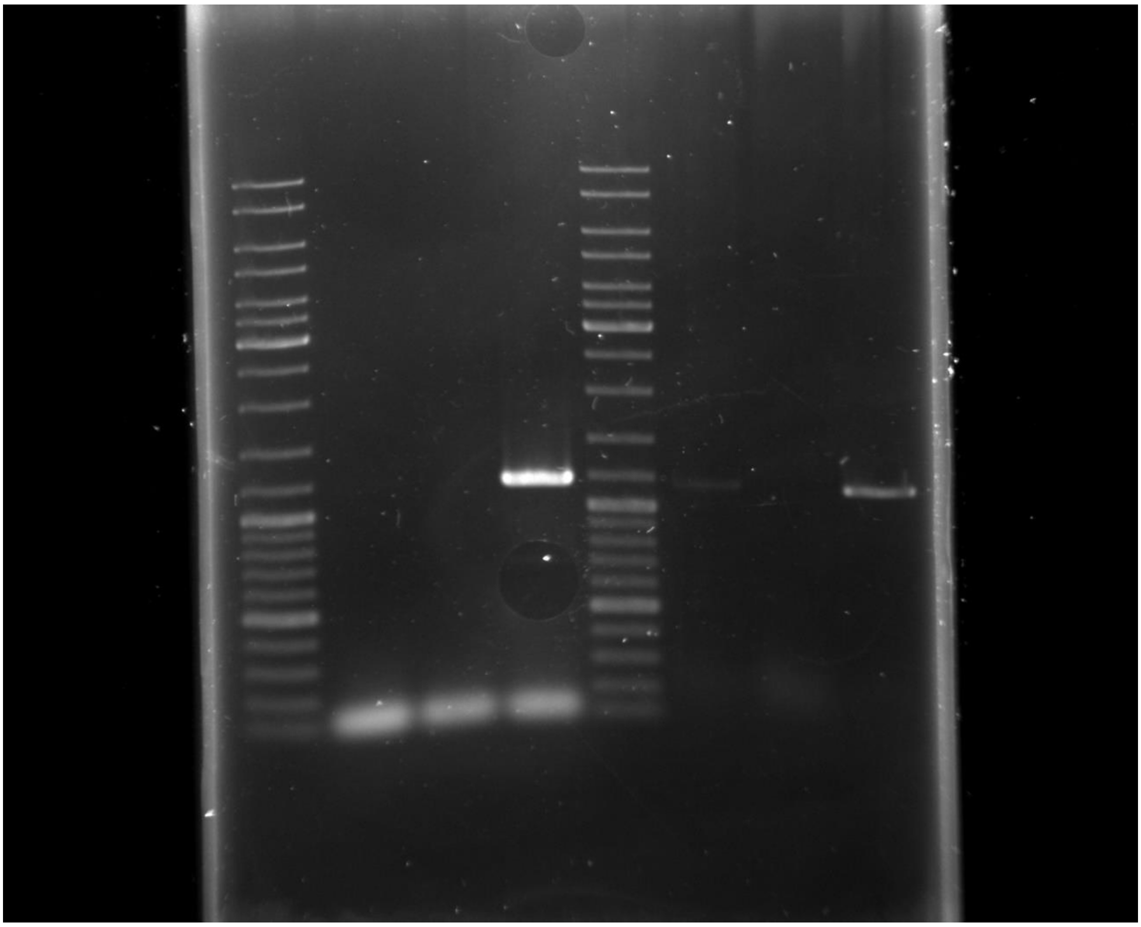

Supplement: Supplementary file 1 — Supplementary material [file 41598_2018_22309_MOESM1_ESM.pdf]
